# LncRNA HOXB-AS4 Promotes Tumor Malignant Phenotype in Head and Neck Squamous Cell Carcinoma and Serves as a Prognosis Marker

**DOI:** 10.7150/jca.111037

**Published:** 2025-07-01

**Authors:** Shanshan Lv, Jie Guo, Lin Du, Yanwei Luo, Hao Tian, Yan Liu

**Affiliations:** 1 Hunan Cancer Hospital and The Affiliated Cancer Hospital of Xiangya School of Medicine, Central South University, Changsha, 410013, Hunan, China.; 2 Department of Blood Transfusion, The Third Xiangya Hospital of Central South University, Changsha, China.; 3 National Institution of Drug Clinical Trial, Xiangya Hospital, Central South University, Changsha, China.; 4 China National Clinical Research Center for Geriatric Disorders, Xiangya Hospital, Central South University, Changsha, China.; 5 Department of Head and Neck Surgery, Hunan Cancer Hospital and The Affiliated Cancer Hospital of Xiangya School of Medicine, Central South University, Changsha, 410013, Hunan, China.

**Keywords:** Head and neck squamous cell carcinoma, HOXB-AS4, HOXB7, AKT, p-AKT

## Abstract

**Background:** Head and neck squamous cell carcinoma (HNSCC) is the most common malignant tumor in the head and neck with a high suicide rate. Numerous studies have indicated that lncRNAs play a significant role in tumor occurrence and development, and that they may serve as promising diagnostic markers and therapeutic targets. The lncRNA HOXB-AS4 is substantially expressed in HNSCC; this work aimed to clarify the role of HOXB-AS4 in HNSCC and to further investigate its potential mode of action.

**Methods:** In this study, bioinformatics analysis was utilized to identify differentially expressed lncRNAs from RNA-seq data in the TCGA-HNSCC data set. The effect of lncRNA on HNSCC cell function was assessed using cell function tests. The probable downstream target mRNA of lncRNA was discovered after analyzing the differential mRNA and reviewing the literature. Mass spectrometry was utilized to investigate the signaling pathways it may control. RT-qPCR and western blotting were employed to confirm its regulatory action.

**Results:** HOXB-AS4 was abnormally overexpressed in HNSCC, which was related with poor clinical characteristics and prognosis, as well as promoting HNSCC cell migration, invasion, proliferation, and clone formation. The elevated expression of HOXB-AS4 in HNSCC may play a role in tumor promotion by influencing the HOXB7 gene located on the same chromosome, thereby activating the phosphorylation of AKT.

**Conclusions:** HOXB-AS4 may promote malignancy in HNSCC by controlling the HOXB7/AKT pathway.

## Introduction

Head and neck squamous cell carcinoma (HNSCC) is the most common malignant tumor in the head and neck, originating from the mucosal epithelium of the oral cavity, nasopharynx, and larynx 1. According to data from The Global Cancer Observatory (GLOBOCAN) for 2020, the incidence and mortality rate of HNSCC, mainly associated with betel nut chewing, smoking, alcohol consumption, bacterial infections, HPV infection, and ultraviolet radiation, have shown higher in South-Central Asia, Eastern Europe, and Western Europe. Additionally, in all regions, the morbidity and mortality rates for men are consistently higher than those for women 2,3. HNSCC not only affects the vital physiological functions of breathing, chewing, and swallowing, but it also predisposes patients to psychological issues due to their appearance. As a result, in addition to direct tumor-related deaths, HNSCC patients have a greater suicide incidence than other tumor patients, second only to pancreatic cancer 4. More than 60% of HNSCC patients are in late stages when they are first diagnosed, with recurrence rates as high as 40-60% after multimodal treatments such as surgery, radiation, and chemotherapy, and patients with distant metastases have an exceedingly low 5-year survival rate 5,6. Nowadays, it is still necessary to research on the putative molecular processes of HNSCC development and metastasis in order to develop novel strategies for early tumor identification and treatment.

Long non-coding RNAs (lncRNAs) are RNAs longer than 200 nt that do not encode proteins and make up a major component of the genomes of complex organisms. Broadly defined lncRNAs include RNAs transcribed by RNA polymerase I (Pol I), Pol II, and Pol III, as well as RNAs generated from processed introns. Now, it has been proposed that non-coding RNAs be divided into three groups to better describe lncRNAs, which are longer than 500 nt and primarily originate from Pol II 7. The number of currently identified lncRNAs is gradually increasing and now surpasses 20,000 8, most of which are functionally unknown, with only a few recognized to regulate a wide range of biological activities via various molecular mechanisms 7. The majority of lncRNAs with better characterizations are linked to the regulation of gene transcription and have many modes of action on transcriptional regulatory processes 9,10. Some lncRNAs interact spatially with neighboring mRNA-expressed genes 11. For example, when released from chromatin-associated transcription sites, the human lncRNA A-ROD enhances the expression of its neighboring protein-coding gene, DKK1 12. Some lncRNAs are also found near transcription factors that act as cis-transcriptional stabilizers 11. For example, the mouse lncRNA Halr1, also known as linc-HOXA1, is located 40kb upstream of the homologous transcription factor gene hoxa1, and its depletion or promoter variation leads to increased expression of hoxa1 or other genes in the hoxa family 13,14. Furthermore, some lncRNAs can function as transcriptional regulators 11.

As lncRNAs are continually being investigated, numerous studies have discovered their involvement in a range of pathological processes, highlighting research topics including cancer 15-17, cardiovascular disease, etc, and about one-third of the papers related with the keyword "lncRNA" in PubMed contain the phrase "cancer" 18. Some of these researches have focused on the function of lncRNAs in HNSCC. For instance, the lncRNA RASAL2-AS1 plays a pro-cancer role in HNSCC by boosting METTL14-mediated methylation of m6A and influencing the downstream target gene LIS1 19. In addition, some other lncRNAs, including LINC01614, LINC00313, and HOXC13-AS, also have significant functions in HNSCC through various modes of action and can be employed as prognostic and diagnostic indicators for HNSCC 20-22. However, the majority of lncRNAs involved in HNSCC have yet to be reported.

The Cancer Genome Atlas Program (TCGA) database has the distinct advantage of being able to research gene expression at a large sample level, thereby facilitating the discovery of new markers and therapeutic targets. At present, there have been some research articles by mining HNSCC data in TCGA database 23. In this study, we employed a large amount of data on lncRNAs from the RNA sequencing data of the TCGA database to identify key molecules in HNSCC through TCGA data mining combined with Lasso regression analysis, and found that HOXB-AS4 is an independent prognostic marker for HNSCC. Further investigation of this molecule may aid in the development of novel diagnostic and therapeutic markers for HNSCC.

## Materials and methods

### Bioinformatics analysis

The TCGA database (http://cancergenome.nih.gov/) provided the RNA-seq data for the HNSCC, and the data were collated and standardized using R software. Download the other two data sets (GSE41613 and GSE42743) from the GEO database (https://www.ncbi.nlm.nih.gov/geo/) as a validation set. The "Limma" package was used to assess differential lncRNA and mRNA between tumor and normal groups. Genes with |log_2_FC| > 5 and p.adj <0.05 were identified as differential genes. The "glmnet" package was used for LASSO regression to build the prognostic model, while the "survival", "ROCR", and "rms" packages were used for survival analysis, ROC curve, and nomogram. The BEST online database (https://rookieutopia.com/app_direct/BEST/) examined the relationship between the expression of HOXB-AS4 and the clinical pathological characteristics of HNSCC patients. Gene ontology (GO) and Kyoto Encyclopedia of Genes and Genomes (KEGG) enrichment analyses were carried out with the "clusterProfiler" program in R software. Mass spectrometry (MS) data were processed using the TOmicsVics program for programmable counter array (PCA) and GSEA software for ‌Gene Set Enrichment Analysis. Immune infiltration analyses were performed using the ASSISTANT for Clinical Bioinformatics online tools (https://www.aclbi.com/static/index.html#/) and TCGA - HNSC data sets. Genomic alteration and pharmacological analyses were performed using the BEST website (https://rookieutopia.com/app_direct/BEST/).

### Cell culture and transfection

The Cal-27 cell line was donated by the Department of Stomatology, the second hospital of Xiangya, the Central South University, which had previously been purchased from the American Type Culture Collection cell bank, and it was cultivated in DMEM (Thermo Fisher, gibco) media containing 10% fetal bovine serum (VivaCell, C04001-500) and 1% penicillin-streptomycin (Thermo Fisher, gibco). The SCC-9 cell line was purchased from Wuhan Pricella Biotechnology Co, and it was cultured in DMEM/F12 (Thermo Fisher, gibco) complete medium containing the same serum and antibiotic. Both cell lines were cultured in an incubator with 5% CO2 at 37°C and proved negative for mycoplasma. Transfection was performed when the cell density reached 60%. To complete the transfection, siRNA was combined with the transfection reagent in opti-MEM (Thermo Fisher, gibco) according to the Lipofectamine 3000 (Thermo Fisher Scientific, L3000015) instructions and then dropped into the cell culture dish. All siRNA was designed and synthesized by Guangzhou RiboBio Co. The sequences of siRNAs targeting HOXB-AS4 were as follows: siRNA-HOXB-AS4-1: GGTGACTCCCAAGGCCTGA, siRNA-HOXB-AS4-2: CGTTTATTGCCGGGTACTT, siRNA-HOXB-AS4-3: CCGTCTCCTTATCTCTTTA.

### Detection of cell proliferation

After 48 hours of cell transfection, cells were digested with 0.25% trypsin-EDTA (Thermo Fisher, gibco) at 37°C for 2min, and terminated by the addition of complete medium with twice the volume of trypsin. The cells were then collected and counted, and a cell suspension was prepared at a concentration of 1×10^4^ cells per ml, then added 200μl per well of the above cell suspension to 96-well plates, and the cells were incubated for 2 hours after the addition of CCK8 reagent (life-ilab, AC11L054) in every day for the next 0-6 days. The absorbance values were then measured using microplate reader, analyzed with GraphPad Prism 8, and plotted on line charts.

### Detection of cell migration and invasion

The transwell assay was used to detect the migration and invasion ability of the cells, we should add the matrix gel (CORNING, 356234) into the chambers (CORNING, 353097) and keep it at 37°C for longer than two hours to make it polymerize into a gel for use. After 48 hours of transfection, cells were prepared into cell suspensions at concentrations of 4×10^4^ cells per ml and 2×10^4^ cells per ml using medium containing 2% FBS, then 200μl per well of above cell suspension was added to the chambers with and without matrix gel respectively to detect cell invasion and migration ability, while 800μl medium containing 15% FBS was added to the lower chamber to create a concentration difference between the upper and lower chambers to enable the cells to better move downward through the chambers. After 24 hours of incubation at 37°C, removed the chambers, washed twice with PBS (Thermo Fisher, gibco), and the cells were fixed with 4% paraformaldehyde (Sangon Biotech, Shanghai) for 15 minutes, followed by staining with crystal violet (beyotime, C0121) for 15 minutes at room temperature, washing off the excess crystal violet, wiping off the stromal gel on the upper surface with a cotton ball, drying and then photographing three fields of view per well under the inverted microscope, observing and counting the number of cells that had passed through the chambers, and analyzing and plotting bar charts using GraphPad Prism 8.

### Clone formation assay

After 48 hours of transfection, cells were prepared into a cell suspension at a concentration of 500 cells per ml using the complete medium, and 2ml of the above cell suspension was added to each well of a six-well plate, i.e., each well contained 1000 cells, and the culture was stopped when the number of individual cell clones reached more than 50 under the microscope. The cells were washed twice with PBS, fixed with 4% paraformaldehyde for 15 minutes, stained with crystal violet staining solution for 15 minutes at room temperature, protected from light, rinsed off the excess crystal violet with running water, dried, and counted using the Adobe Illustrator 2022 APP technology tool, then analyzed and plotted on bar charts using GraphPad Prism 8.

### Quantitative Real-time PCR

Tumors and paracancerous tissues were collected from the Department of Head and Neck Surgery, Hunan Cancer Hospital. The conventional trizol method was used to extract RNA from tissue and cell samples. The RNA concentration was then determined and reverse-transcribed into cDNA in accordance with the instructions provided by the Reverse Transcription Kit (Roche). Real-time fluorescence quantitative PCR (RT-qPCR) was used to perform relative quantitative assays. The system consisted of 2 μl of the cDNA template, 1 μl of each upstream and downstream primer, 1 μl of water, and 5 μl of a 2× SYBR GREEN (Roche). RT-qPCR relative quantitative detection was performed using the LightCycler 480 II (Roche). The target gene's relative expression was calculated using β-actin as an internal reference. Q-PCR primers used were all synthesized by Sangon Biotech, and the sequences were: HOXB-AS4: forward 5'-TTTGTCTGCTCTGGACCGTT-3' and reverse 5'-TTTTGGTTCGGAGGCCTAGT-3'; β-actin: forward 5'-TCACCAACTGGGACGACATG-3' and reverse 5'-GTCACCGGAGTCCATCACGAT-3'; HOXB7: forward 5'-AGGAACTGACCGCAAACGAG-3' and reverse 5'-ATCTGTCTTTCCGTGAGGCA-3'; HOXB9: forward 5'-GGAGGCCGTGCTGTCTAATC-3' and reverse 5'-GATCCGGCCTCTCTTTGTCC-3'.

### Western blot

After 72 hours of transfection, cell precipitates were collected, proteins were extracted using RIPA lysate (Beyotime, P0013E) supplemented with protease inhibitors, determined the protein concentration in accordance with the guidelines provided by the BCA Protein Concentration Assay Kit (Thermo Fisher, 23225), and computed the sample volume. After SDS-PAGE electrophoresis (Epizyme, PG212), the protein bands were transferred to the PVDF membrane (Merck Millipore, IPVH00010), which was then closed at room temperature for 1 hour with 5% skimmed milk powder, then immersed in the primary antibody at a dilution of 1:1000 and set at 4°C overnight. The PVDF membrane was washed in TBST the next day, and the corresponding secondary antibody was chosen based on the species of the primary antibody for incubation at room temperature for 1 hour, followed by another TBST wash, and color was developed on a chromatograph using the ECL Ultra Sensitive Chromatography Reagent (Thermo Fisher, A38554). Adobe Photoshop 2023 software was used to process and create the photographs. The main primary antibodies employed in the experiment were: AKT (proteintech, 10176-2-AP), Phospho-AKT (proteintech, 28731-1-AP), HOXB7 (ABclonal, A6925) Alpha Tubulin (proteintech, 11224-1-AP), GAPDH (Beijing ComWin Biotech, 01225); and secondary antibody were: Goat Anti-Rabbit IgG, HRP Conjugated (Beijing ComWin Biotech, 01334), Goat Anti-Mouse IgG, HRP Conjugated (Beijing ComWin Biotech, 01123).

### Immunofluorescence

After 48 hours of cell transfection, the cells were allowed to form a monolayer of adherent cells on the cell crawler sheet at a density of roughly 60%. The cells on the crawler sheet were washed twice with PBS, fixed with 4% paraformaldehyde at room temperature for 15 minutes, washed, for this experiment is used to detect cytoskeletal molecules, it needs to use the immunostaining permeabilization solution Triton X-100 (Beyotime, P0096) to permeabilize at room temperature for 5 minutes, moisten with PBS for three times, and then block with immunostaining blocking solution (Beyotime, P0102) for 1h after permeabilization, and a primary antibody was added dropwise to the crawler sheet. The usual dilution ratio was 1:50, and the temperature was set to 4°C for overnight treatment. The film was washed twice with PBST the next day after rewarming, and the corresponding fluorescent secondary antibody was applied based on the species of primary antibody. After 1 hour of incubation at room temperature and light protection, the film was dyed with DAPI-containing sealer (Beyotime, P0131) and sealed. Each slide was photographed under the fluorescence microscope in 5 different fields of view at random. The primary antibodies employed in the experiment were: CL594-Phalloidin (Proteintech, PF00003), and fluorescent secondary antibodies were: AF488 donkey anti-mouse IgG (H+L) (Thermo Fisher 1975519).

### Statistical analysis

The experimental results were analyzed with GraphPad Prism software (version 8.0; La Jolla, CA, USA). The differences between the two groups were calculated using t-tests. Significant differences between several data sets were calculated using a one-way analysis of variance (ANOVA). p < 0.05 indicated statistical significance. * p <0.05, ** p <0.01, *** p <0.001.

## Results

### Screening differentially expressed lncRNAs and building a prognostic model for HNSCC-associated lncRNAs

**Figure 1:**
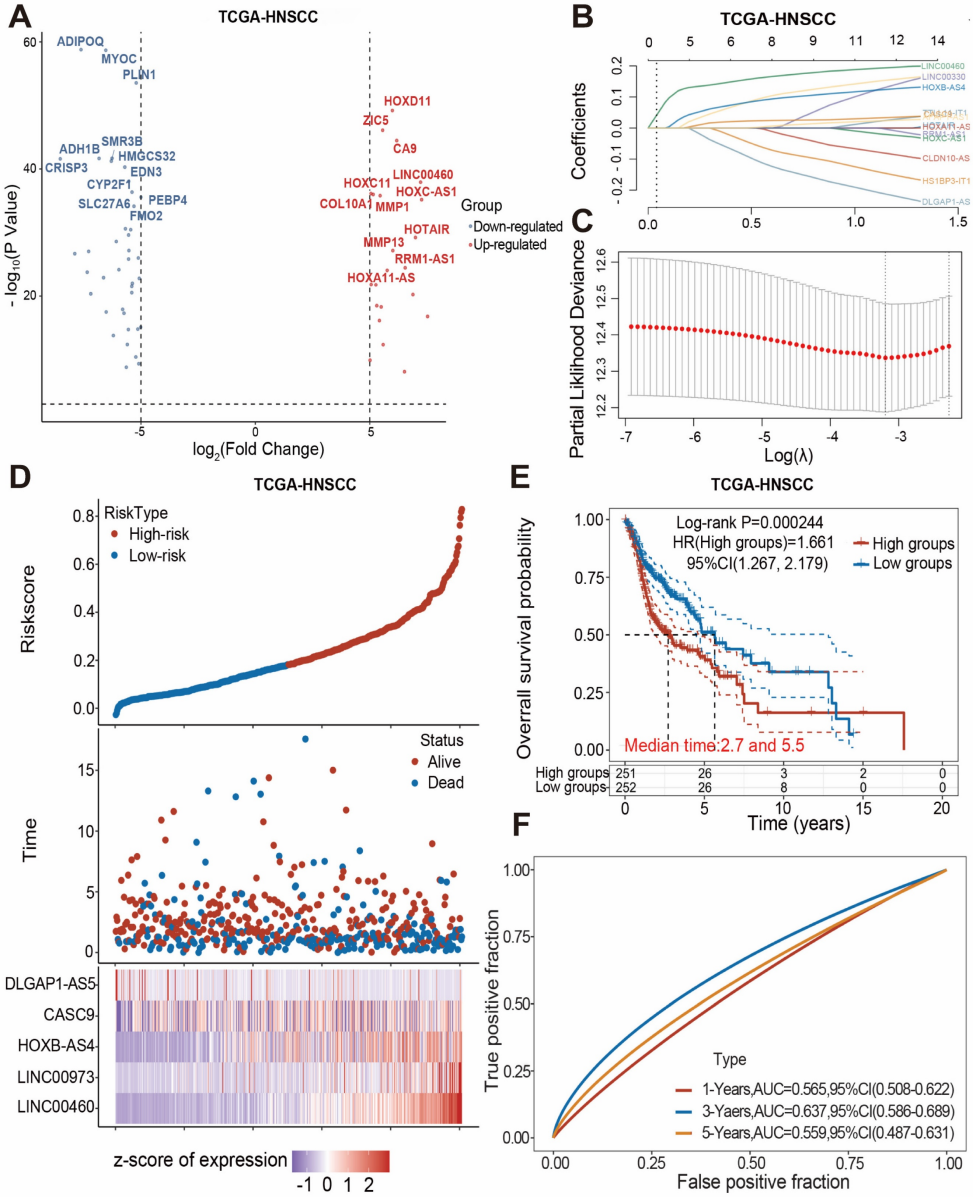
** Building a prognostic model for HNSCC-associated lncRNAs.** (A) Volcano plots showing differential expression of lncRNAs between HNSCC and normal control groups. (B-C) Lasso regression screening and construction of prognostic model consisting of 5 lncRNAs. (D) Distribution of patient survival status between high- and low-risk groups and risk scores for the 5 lncRNAs. (E) Kaplan-Meier survival analysis between high- and low-risk groups. (F) The ROC curve shows the prediction probability of the prognostic model.

The transcriptome data for HNSCC were obtained from the Cance Genome Atlas (TCGA) database, and the differentially expressed genes between the cancer group and the normal group were obtained by comparing the transcriptome data of these two groups according to the principle of |log_2_FC| > 5, and all differentially expressed genes were visualized using volcano plots (Figure 1A).

The blue dots on the left side represent differentially down-regulated genes in comparison to the control group, while the red dots on the right side represent differentially up-regulated genes. According to the data that combining the differentially expressed lncRNAs with the clinical prognostic information of patients in the TCGA database, five lncRNAs (DLGAP1-AS5, CASC9, HOXB-AS4, LINC00973, LINC00460) were screened using Lasso regression and constructed the HNSC-associated prognostic model (Figure 1B-C). The HNSCC patients in the TCGA database were divided into high-risk and low-risk subgroups based on the median risk score. As shown in Figure 1D, the high-risk group had a larger proportion of deaths, and HOXB-AS4, LINC00973, and LINC00460 had higher risk ratings in that group. Kaplan-Meier survival analyses revealed that risk ratings were related to HNSCC prognosis, with patients in the high-risk group having a worse prognosis (Figure 1E). ROC curves revealed that the prognostic risk model predicted 1-year, 3-year, and 5-year survival with AUC values of 0.565, 0.637, and 0.559, respectively (Figure 1F). The effect of the above prognostic model was validated using two external datasets, GSE41613 and GSE42743. The Kaplan-Meier survival analysis results confirmed the TCGA database results (Supplementary Figure 1), indicating that the prognostic model was more stable.

### LncRNA HOXB-AS4 has better prognostic marker value in HNSCC

**Figure 2:**
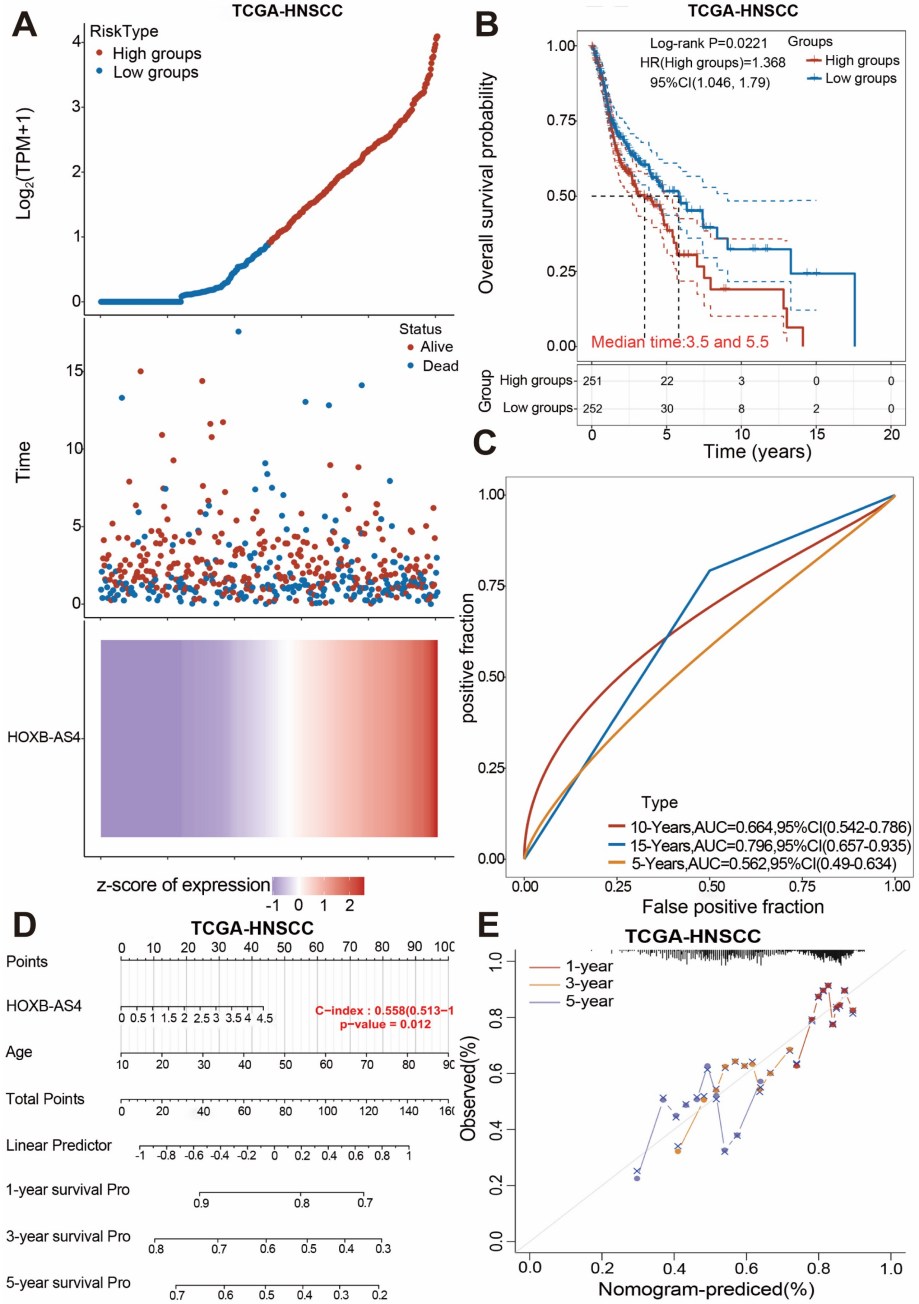
**The prognostic value analysis of HOXB-AS4 in HNSCC.** HNSCC patients were classified to high and low group according to their median HOXB-AS4 risk score. (A) Grouping, survival status distribution, and the HOXB-AS4 risk score. (B) Kaplan-Meier survival analysis. (C) ROC curve. (D-E) Nomogram and calibration curves.

The human HOX family is divided into four subfamilies: A, B, C, and D, which are found on chromosomes 7,17,22,2, with a total of 39 members 24. HOX genes are overexpressed in a variety of tumors and are strongly related to tumor stage and prognosis 25. The analysis results in Figure 1 showed that HOXB-AS4 had a higher risk score in the cancer group, so we hypothesized that HOXB-AS4 might play a potential role in HNSCC. HNSCC patients were divided into two groups based on the median value of the HXOB-AS4 risk score, with 251 patients in the high-risk group and 252 patients in the low-risk group, and the survival distribution status of patients in the two groups revealed that the high-risk group had more deaths (Figure 2A). The Kaplan-Meier survival analysis showed that HOXB-AS4 was associated with the prognosis of HNSCC, and patients in the high-risk group had a poorer prognosis (Figure 2B). The ROC curves showed that the AUCs for predicting 5-year, 10-year, and 15-year survival based on HOXB-AS4 were 0.562,0.664, and 0.796, respectively (Figure 2C). A nomogram based on HOXB-AS4 expression level and age was created to assess the survival probability of HNSCC (Figure 2D), while the results of calibration curves showed that the predicted results of the nomogram were more consistent with the actual results (Figure 2E). The predictive effect of HOXB-AS4 in HNSCC was confirmed by Kaplan-Meier survival analyses of external datasets GSE41613 and GSE42743 (Supplementary Figure 2).

### HOXB-AS4 is associated with worse clinicopathological features in HNSCC patients

**Figure 3:**
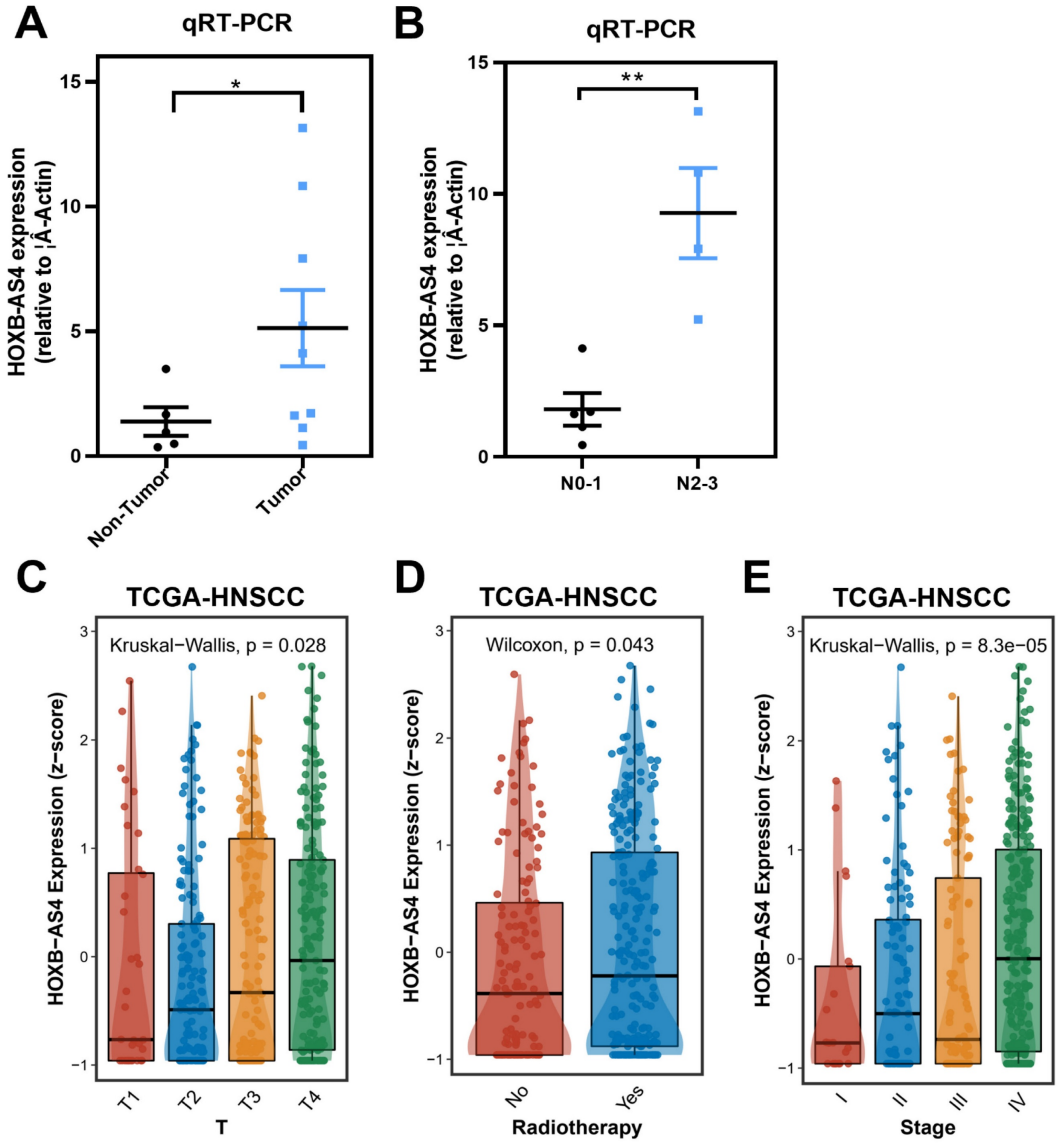
**Correlation analysis between HOXB-AS4 and clinicopathological characteristics of HNSCC patients.** (A) RT-qPCR results from HNSCC patient tissue samples. (B) Comparison of HOXB-AS4 expression in patients with different N stages. (C-E) The relationship of HOXB-AS4 to T stage (C), radiotherapy (D), and total patient stage (E).

Tumor and paracancerous tissues of HNSCC patients were acquired from Hunan Cancer Hospital, and RT-qPCR validated the expression of HOXB-AS4 after extracting the tissue RNA. The results showed that it was much higher in tumor tissues than paracancerous tissues (Figure 3A). While we had also collected their N stage from some of these patients, and divided them into N0-1 AND N2-3 groups according to their N stage, the comparison result showed that the expression of HOXB-AS4 was higher in the more severe N stage group (Figure 3B).

The clinical information of HNSCC patients was downloaded from the TCGA database and the analytic results of the correlation between HOXB-AS4 expression and pathological features were shown in Figure 3C-E, patients who received radiotherapy had higher HOXB-AS4 expression than those who did not, and in the analysis of the T staging, the level of HOXB-AS4 expression increased gradually with the increase of the T staging, and in total clinical staging it was similarly observed that HOXB-AS4 expression was higher in more advanced tumor stages. These results suggest that HOXB-AS4 expression is up-regulated in HNSCC, which predicts a worse clinical stage and prognosis.

### Silencing of HOXB-AS4 inhibits the ability of HNSCC metastasis and proliferation but does not act by regulating the cytoskeleton

**Figure 4:**
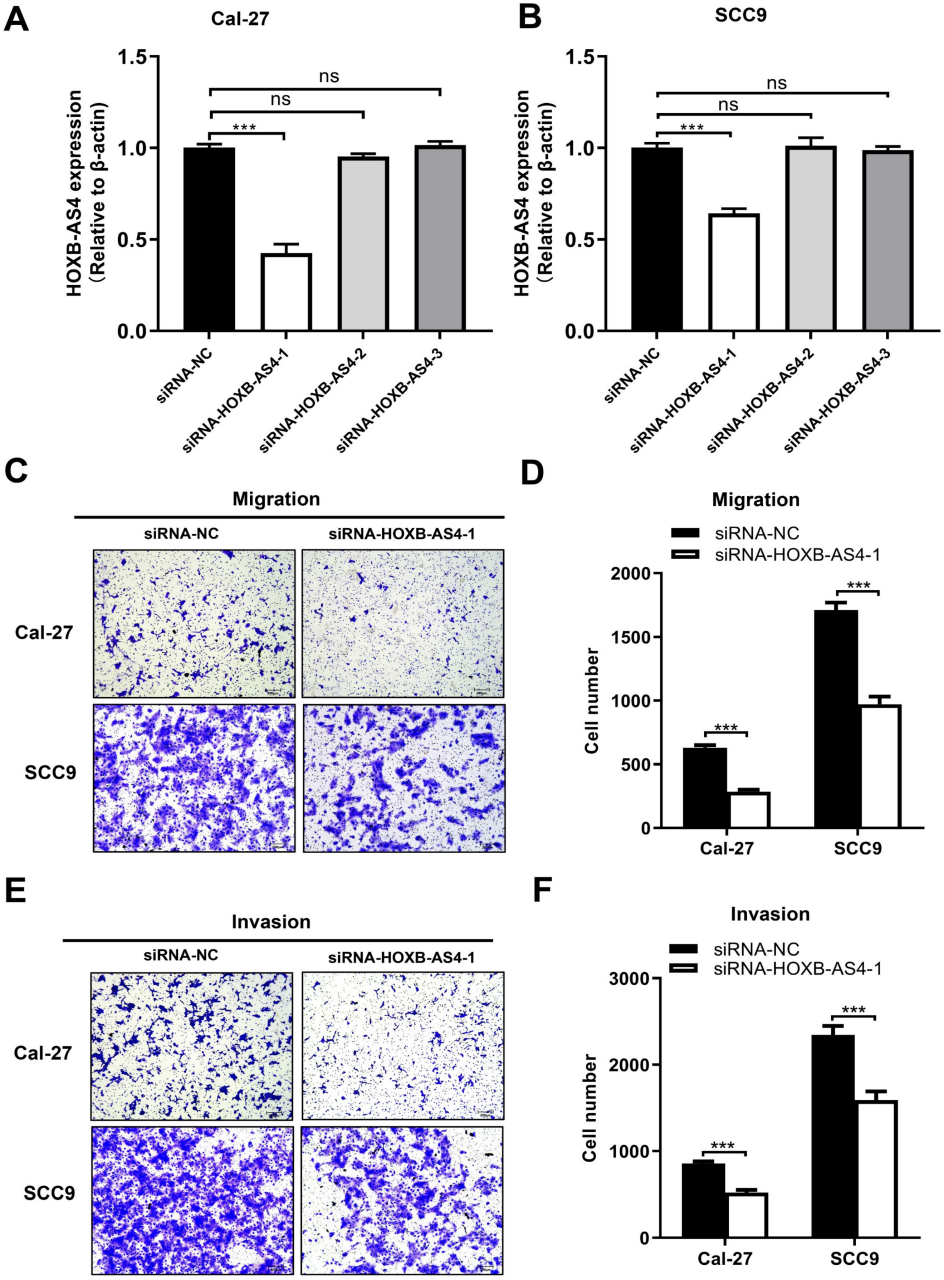
**Effect of HOXB-AS4 on HNSCC cell migration and invasion.** (A-B) The silencing impact of HOXB-AS4 siRNAs was detected by using RT-qPCR. (C) Transwell cell migration test to assess the effect of silencing HOXB-AS4 on HNSCC cell movement. (D) A statistical map of HNSCC cell movement following HOXB-AS4 silencing. (E) Transwell cell invasion test to assess the effect of silencing HOXB-AS4 on HNSCC cell invasion. (F) A statistical map of HNSCC cell invasion following HOXB-AS4 silencing.

**Figure 5:**
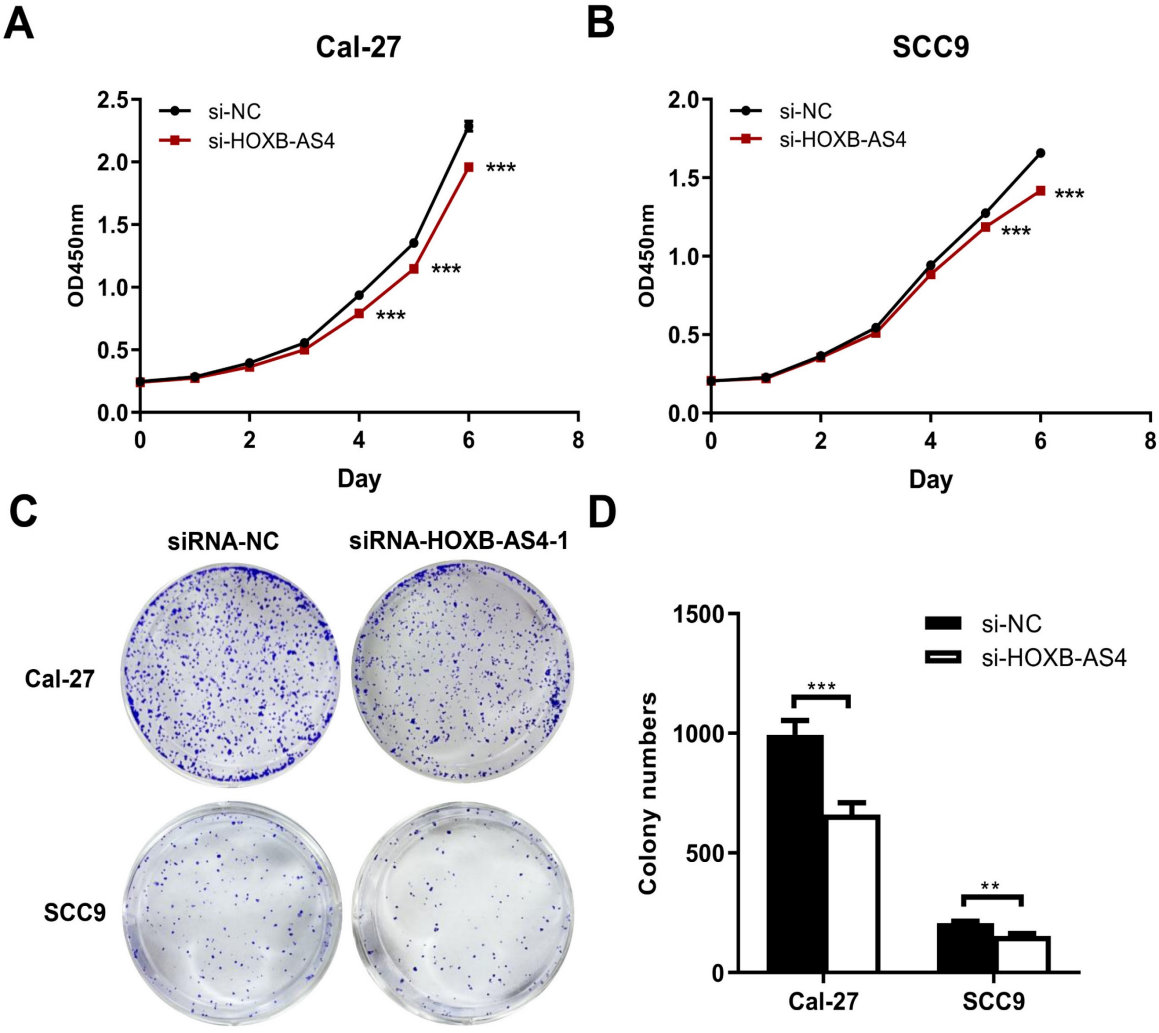
**Effect of HOXB-AS4 on HNSCC cell proliferation and clonogenesis.** (A-B) The cck8 cell proliferation test to assess the effect of HOXB-AS4 silencing on HNSCC cell proliferation. (C)The clonal formation test to assess the effect of HOXB-AS4 silencing on the clonal ability of HNSCC cells. (D) A statistical map of HNSCC cell clonal formation following HOXB-AS4 silencing.

HOXB-AS4 in Cal-27 and SCC-9 cell lines was silenced using siRNA, and the effectiveness of the silencing was verified by RT-qPCR. As demonstrated in Figure 4A-B, hence it was chosen for the following tests. Silencing HOXB-AS4 significantly reduced the migration (Figure 4C-D) and invasion (Figure 4E-F) abilities of Cal-27 and SCC-9 cells, and the results of cck8 and clone formation experiments revealed that the two cell lines' proliferative and clone formation abilities were also reduced (Figure 5A-D). In summary, silencing HOXB-AS4 was able to inhibit the migration, invasion, proliferation, and clone formation ability of HNSCC cells.

To further explore the possible mechanisms by which HOXB-AS4 contributes to the malignant phenotype of HNSCC, we performed GO (S3A) and KEGG (S3B) enrichment analyses on both HOXB-AS4 high- and low-expressing subgroups and found that significant enrichment was observed in the cellular components assembly involved in morphogenesis, as well as regulation of actin cytoskeleton and focal adhesion. However, cellular immunofluorescence staining found no significant difference in the cytoskeleton and nucleus co-staining (S3C) of cells from control and silencing groups. This result suggests that HOXB-AS4 may also not function by regulating the cytoskeleton.

### HOXB-AS4 may affect the AKT signaling pathway by regulating HOXB7 expression leading to a malignant phenotype in HNSCC cells

**Figure 6:**
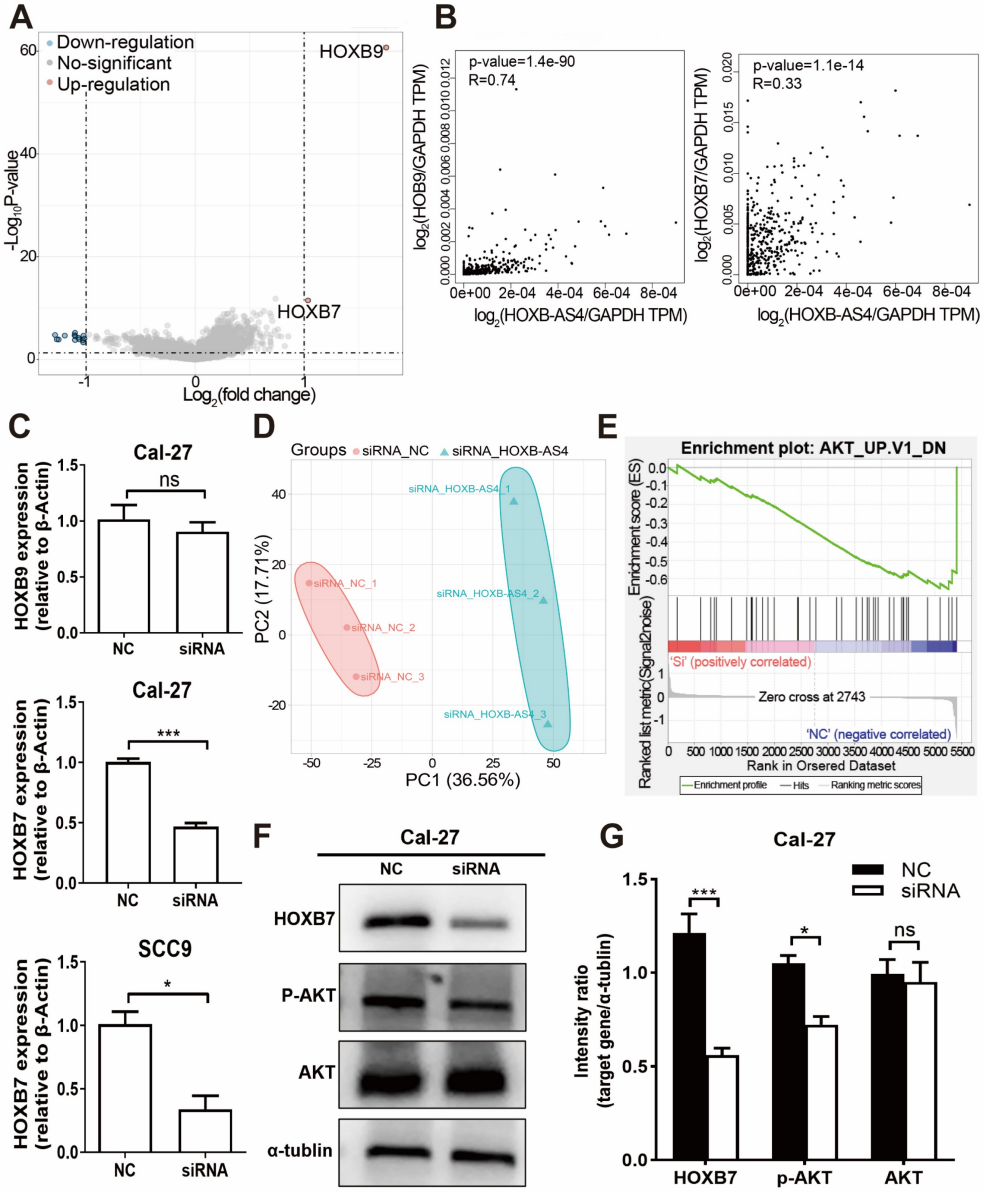
**HOXB-AS4 may activate the AKT signaling pathway by regulating HOXB7, resulting in malignant phenotypes.** (A) Comparison of HNSCC mRNA differences in the TCGA database. (B) Correlation analysis of HOXB-AS4 and HOXB7 or HOXB9. (C) RT-qPCR confirmed the expression of HOXB7 and HOXB9 in Cal-27 cells after knocking down HOXB-AS4. (D) PCA score map for proteomic data. (E) GSEA enrichment analysis enhanced the differential AKT signaling pathway. (F) Western blot analysis indicated changes in HOXB7, AKT, and p-AKT levels. (G) The quantification diagram of the western blot results.

Downloading the mRNA sequencing data from the TCGA database for differential analysis revealed that HOXB7 and HOXB9, which are located at different positions on the same chromosome as HOXB-AS4, were up-regulated in the tumors (Fig. 6A). Additionally, the correlation analysis showed a positive correlation between HOXB-AS4 and the expression of both HOXB7 and HOXB9 (Fig. 6B). After silencing HOXB-AS4 using siRNA transfection of Cal-27 cells, the cells were collected for RNA extraction, reverse transcribed, and then analyzed by RT-qPCR to detect the expression levels of HOXB7 and HOXB9. The results showed that HOXB7 expression was reduced compared with the control group, while HOXB9 expression was not significantly different. In SCC9 cells, the expression of HOXB7 is also reduced after the knockdown of HOXB-AS4 (Figure 6C). Therefore, we hypothesized that HOXB-AS4 may act by affecting the mRNA HOXB7 of the same family on the same chromosome. Cell samples transfected with siRNA were collected and divided into experimental and control groups, each containing three biological replicates, for proteome analysis using liquid chromatography-tandem mass spectrometry (LC-MS). The scores were shown using principal component analysis (Fig. 6D), and it was discovered that the control and experimental samples were clearly separated from each other with large differences. The proteome data were then subjected to GSEA enrichment analysis to search for potential downstream pathways, and the disease was significantly enriched to the differentiated AKT signaling pathway (Figure 6E). Cal-27 cell samples were collected for protein extraction, and the results of western blot experiments showed that silencing of HOXB-AS4 decreased HOXB7 protein expression and AKT phosphorylation level (Figure 6F). Figure 6G is the quantification diagram of the western blot results. Based on the foregoing analysis and experimental data, we hypothesized that lncRNA HOXB-AS4 may alter the AKT signaling pathway by regulating mRNA HOXB7, resulting in the formation of a malignant phenotype and a poor prognosis in HNSCC.

### HOXB-AS4 is related to HNSCC tumor immunity, stemness, and treatment resistance

**Figure 7:**
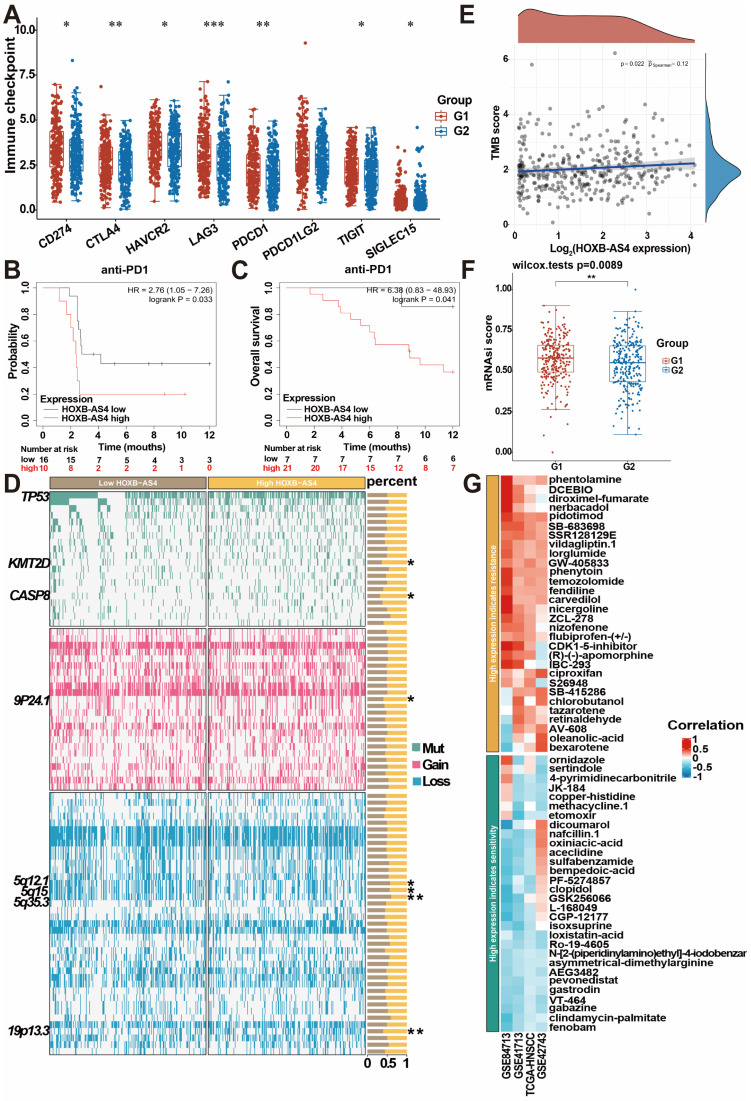
**HOXB-AS4 plays a role in HNSC tumor immunity, stemness, and drug sensitivity.** (A) Differential analysis of major immune checkpoints in the TCGA database. (B-C) Effect of HOXB-AS4 expression on survival probability and overall survival after anti-PD-1 immunotherapy. (D) Genomic alteration analysis. (E) Tumor mutational burden. (F) Tumor cell stemness index. (G) Drug sensitivity analysis.

HNSCC patients in the TCGA database were divided into two groups, G1 (high HOXB-AS4 expression) and G2 (low HOXB-AS4 expression), based on the expression of HOXB-AS4, and analyzed potential differences in immune checkpoints between the two groups. The results indicated that the high HOXB-AS4 expression group exhibited elevated levels of immune checkpoints such as CD274, CTLA4, HAVCR2, LAG3, PDCD1, TIGIT, and SIGLEC15 compared to the low expression group (Figure 7A). This suggests that HOXB-AS4 may contribute to a pro-tumor effect by promoting immune escape in tumor cells. However, survival analysis revealed that patients in the high-expression group with anti-PD1 immunotherapy had poorer survival probability and overall survival rates than patients in the low-expression group also with anti-PD1 immunotherapy (Figure 7B-C). It is speculated that the reason for this may be due to the fact that high HOXB-AS4 expression also upregulates other immune checkpoints such as CTLA4, HAVCR2, LAG3, etc., which may play alternative roles and continue to promote tumor immune escape after PD1 blockade on the one hand, and on the other hand, it is possible that the complex tumor microenvironment containing various immune cells and signaling pathways collectively impact immune inhibitor efficacy. Genomic alteration analysis was conducted to compare mutations, amplifications, and deletions of genes in the HOXB-AS4 high-expression group with those in the low-expression group. The results revealed an increase in KMT2D and CASP8 mutations, which are related to the tumor immune microenvironment, as well as an amplification of chromosome 9p24.1, and deletions of chromosomes 5q12.1, 5q15, and 5q35.3 in the HOXB-AS4 high-expression group (Figure 7D). Tumor Mutation Burden (TMB) analysis indicated a positive correlation between HOXB-AS4 and TMB scores to some extent (Figure 7E). Moreover, mRNAsi score results showed that gene expression levels of tumor stem cells were higher in patients with high expression of HOXB-AS4 (Figure 7F), suggesting that HOXB-AS4 may enhance tumor stem cell properties and promote tumor progression, metastasis, and drug resistance in HNSCC. Pharmacological sensitivity analysis of the GDSC drug database for heatmapping demonstrated a significant positive correlation between high expression of HOXB-AS4 and resistance to certain chemotherapeutic drugs such as phentolamine, DCEBIO, diroximel-fumarate, nerbacadol, pidotimod, SBR128129E, vildagliptin, lorglumide, GW405833, phenytoin, temozolomide, fendiline, carvedilol, nigergoline, ZCL278, nizofenone, flurbiprofen-(+/-), CDK1-5-inhibitor, (R)-(-)-apomorphine, IBC293, ciproxifan, S26948, SB415286, chlorobutanol, tazarotene, retinaldehyde, AV-608, oleanolic-acid and bexarotene (Figure 7G).

## Discussion

LncRNAs were once thought to be useless 'background noise' in the genome 26, but with continued exploration of lncRNAs, it has been discovered that lncRNAs play important and indispensable roles in the whole process of gene expression, including, but not limited to, chromosome modification, epigenetic regulation, transcriptional regulation, and translational regulation, etc. 27-31. Through these regulatory functions, lncRNAs are involved in various biological processes including cell proliferation, cell cycle, apoptosis, and immune response 32-35. Furthermore, numerous studies have shown that abnormal expression of lncRNAs plays a crucial role in the development of various tumors 36. For example, LINC00853 was significantly up-regulated in gastric cancer patients' tissues and promoted the malignant progression of gastric cancer through the MAP17/PDZK1/AKT signaling pathway 37; lncRNA VPS9D1-AS1 was highly expressed in tissue samples of patients with endometrial carcinoma (EC) and was demonstrated to play a pro-cancer role in endometrial cancer by negatively regulating miR-187-3p and positively regulating S100A4 endometrial cancer 38; LINC00313 is highly expressed in HNSCC and affects the progression of HNSCC by influencing the process of epithelial-mesenchymal transition, and the expression of LINC00313 is also negatively correlated with the infiltration of immune cells 20. In our study, we found that the lncRNA HOXB-AS4 was significantly overexpressed in HNSCC by analyzing the data from the TCGA database along with the tumors and normal tissues of patients with HNSCC, and functional experiments confirmed that its high expression correlated with the progression of the malignant phenotype of HNSCC. HOXB-AS4 might be an important marker for predicting the prognosis of HNSCC.

The action mechanism of lncRNA is extremely complex and has not been completely clarified until now. However, numerous studies on the mechanism of lncRNA have been published. First, lncRNAs can be regulated at the epigenetic level by modifying RNA or DNA methylation and histone modification. For example, the lncRNA HOTAIR, which is expressed in the HOXC locus, suppresses HOXD gene expression by directing the H3K27 methylase Polycomb repressive complex 2 (PRC2) complex to the HOXD locus and then modifying chromatin modification in the region. As a result, it accelerates the malignant progression of metastasis and recurrence during carcinogenesis 39,40. Second, lncRNAs can play the regulation both during and after transcription. For example, lncRNA ELDR is closely located downstream of epidermal growth factor (EGF), which is highly expressed in various cancers such as HNSCC and ovarian cancer, and activates the downstream molecule STAT3 by stabilizing EGF, thus ELDR plays a carcinogenic role and serves as a prognostic marker for various cancers 41-43. Furthermore, lncRNAs can compete with miRNA for the mRNA binding site, a process known as competing endogenous RNAs (ceRNAs), inhibiting the regulation of miRNA for mRNA. For example, lncRNA-CDC6 can serve as the ceRNA of miR-215. It also impacts the expression of mRNA CDC6, which promotes breast cancer growth and metastasis, and it might be a predictive biomarker 44. In our study, through the difference analysis of mRNA data of the HNSCC tumor and normal group in the TCGA database, it was found that HOXB7 and HOXB9, located on the same chromosome of HOXB-AS4, were significantly different. RT-qPCR results showed that the expression of HOXB7 was down-regulated after knocking down the HOXB-AS4, and the correlation analysis between them showed a positive correlation. These results suggest that HOXB-AS4 may play a role in promoting cancer in HNSCC by influencing the expression of HOXB7.

HOXB7, which is located on human chromosome 17, is a key regulatory gene that can activate a range of carcinogenic pathways by influencing a variety of target molecules 45. HOXB7 has been found to be overexpressed in a number of tumor tissues and is a valuable early diagnostic and prognostic marker. For example, HOXB7 is a carcinogenic factor that promotes gastric cancer development. Abnormally high HOXB7 expression in gastric cancer is associated with patient clinical characteristics, enhanced the ability of gastric cancer cells to proliferate, migrate, and invade, while inhibiting apoptosis, by activating the PI3KR3/AKT signaling pathway or up-regulating p-AKT and down-regulating PTEN 46. In addition to inducing AKT signaling pathway, it can also promote the proliferation of gastric cancer cells by inducing the expression of MARKs 47. HOXB7 is also overexpressed in colorectal cancer, which can activate the PI3K/AKT and MAPK pathways. It also plays a carcinogenic role in colorectal cancer, making it an essential prognostic marker 48. Furthermore, HOXB7 is overexpressed in solid tumors such as esophageal cancer, intrahepatic cholangiocarcinoma, cutaneous squamous cell carcinoma, osteosarcoma, and pancreatic cancer, suggesting a cancer-promoting role 49-53. A recent study using pan-cancer computer screening found that HOXB7 has value as a pan-cancer marker, and its expression in malignant tumors is also positively correlated with immune cell infiltration, immune regulatory genes, immune checkpoints, and TMB, which may be a potential immunotherapy indicator 54. HOX proteins typically function by attaching to cofactors, such as HOXB7 in melanoma, which relies on the cofactor PBX to play a carcinogenic role 55. Furthermore, HOXB7 can also play an important role in mutual regulation with microRNAs 56,57. In our work, HOXB7 expression was down-regulated in HNSCC cell lines following the silencing of HOXB-AS4 expression. The results of mass spectrometry revealed that the AKT signaling pathway was significantly enriched, and the western blot experiment confirmed that when HOXB-AS4 was down-regulated, p-AKT was also significantly reduced.

The research on the mechanism of HOXB-AS4 in tumors includes: First, through bioinformatics analysis and the characteristics of lncRNA for model prediction, the prognostic value of HOXB-AS4 has been identified in two types of tumors, namely laryngeal squamous cell carcinoma (LSCC) and clear cell renal cell carcinoma (ccRCC), and in ccRCC, HOXB-AS4 is involved in ferroptosis and oxidative stress processes 58-60. Second, in colorectal cancer (CRC), HOXB-AS4 competes with HDAC7 to bind miR-140-5p, thereby promoting the proliferation and migration of CRC cells through the ceRNA regulatory network pathway 61. Therefore, to date, only one study has investigated the role of HOXB-AS4 in HNSCC. However, there are many studies exploring the role of lncRNA in the occurrence and progression of HNSCC. For example, LINC00313 may promote the migration of HNSCC cells by regulating the EMT pathway in HNSCC and is related to immune infiltration 62; by binding to USF1, LINC00152 can enhance the transcription of MRPL52 mediated by USF1, indirectly up-regulating the expression of MRPL52 and promoting the growth of oral squamous cell carcinoma (OSCC) 63; lncRNA FOXD1-AS1 can positively regulate the expression of FOXD1 through miR-369-3p and also increase the stability of FOXD1 mRNA by binding to ADAR protein together with FOXD1, thereby promoting the progression of OSCC through these two mechanisms 64; lncRNA TPRG1-AS1, mediated by CCND1 expression, affects the EMT pathway through the miR-363-3p/MYO1B axis and plays a pro-cancer role in HNSCC 65, and other lncRNAs that act through the ceRNA network pathway in HNSCC include HOXC13-AS and SNHG1 66,67. In addition, most studies have mainly constructed prognostic models of HNSCC-related lncRNAs through model prediction 68-73. In our study, we first discovered that HOXB-AS4 is highly expressed in HNSCC and can promote the malignant progression of HNSCC cells. In terms of mechanism exploration, we found the role of the HOXB-AS4/HOXB7/AKT axis. Thus, HOXB-AS4 may be a valuable prognostic biomarker in HNSCC, and the HOXB-AS4/HOXB7/AKT axis may be a potential therapeutic target for HNSCC.

Immune checkpoints play a crucial role in the self-regulation of the immune system, contributing to autoimmune tolerance under normal physiological conditions. They achieve this process by inhibiting T-cell responses or promoting T-cell depletion through binding to specific ligands, thus effectively terminating the immune response promptly 74. However, in the tumor microenvironment, there is a potential for overexpression of immune checkpoints, leading to immune escape of tumor cells by inhibiting the immune response and ultimately promoting the proliferation and metastasis of tumor cells 75. For example, the programmed cell death protein 1 (PD1) receptor, which can be expressed on the surface of tumor-infiltrating lymphocytes, binds to PD1 ligands on the surface of tumor cells (including PD-L1 and PD-L2, usually PD-L1), transmits negative regulatory signals, and causes apoptosis or loss of function of effector T cells that exert anti-tumor effects 76-78. The cytotoxic T lymphocyte-associated antigen 4 (CTLA4) is the first clinically targeted immune checkpoint receptor expressed only in T cells. When the T-cell receptor (TCR) recognizes an antigen, CD28 amplifies the TCR signal and delivers signals to activate T cells by connecting to the ligands CD80 and CD86. CTLA4, which has the same ligand as CD28, inhibits T-cell activation by competing for binding to the same ligand 79. The role of immunological checkpoints in tumor immunity is complex and needs further investigation. In addition, Lymphocyte activation gene 3 (LAG3) and T-cell membrane protein 3 (TIM3) are promising targets for inhibitors 80,81. Currently, drugs developed against critical immune checkpoints are in clinical trials or are already being used in clinics as immune checkpoint inhibitors, which have become a significant tool in immunotherapy for malignancies 82,83. However, response to single immunosuppressant therapy has been uneven; combinations with multiple immune checkpoint inhibitors or immunotherapy with targeted therapy have proven to be much more effective than single therapies 84,85. In our study, high expression of HOXB-AS4 in HNSCC was associated with high expression of multiple immune checkpoints; however, there was no significant efficacy observed with anti-PD1 therapy. This suggests that a combination approach using multiple immune checkpoint inhibitors may be more effective.

At present, there are still shortcomings in our study. Due to the limitation of clinical sample size, the results of database analysis and cell experiment could not be well combined with more clinical data, so as to strengthen the potential value of HOXB-AS4 as a prognostic marker.

## Conclusion

Based on bioinformatics analysis and experimental verification, our study verified that HOXB-AS4 was overexpressed in HNSCC, which can increase cell proliferation, migration, and invasion. We hypothesized that the function of promoting the malignant progression of HNSCC tumors might work by up-regulating HOXB7 and activating the AKT signaling pathway.

## Supplementary Material

Supplementary figures.

## Figures and Tables

**Figure 8:**
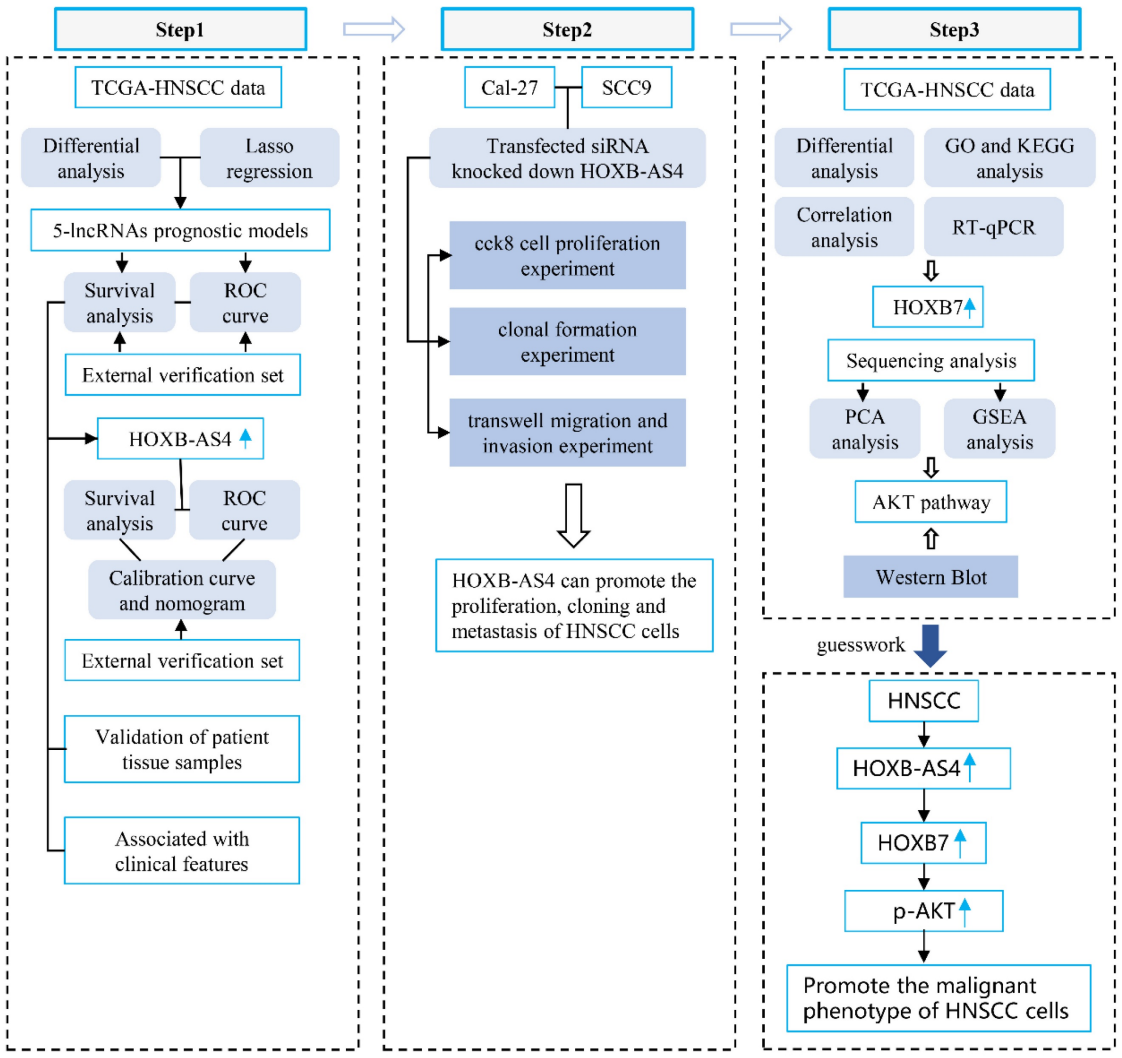
**Flowchart**.
