# Supplementary material for: LncRNA HOXB-AS4 Promotes Tumor Malignant Phenotype in Head and Neck Squamous Cell Carcinoma and Serves as a Prognosis Marker
Source: J Cancer. 2025 Jul 1;16(9):2997–3014. doi: 10.7150/jca.111037 (PMC12244335; doi:10.7150/jca.111037)

S1. Validation of a risk prognostic model consisting of 5 lncRNAs using external datasets. (A-B) Kaplan-Meier survival analysis between high- and low-risk groups in GSE41613 and GSE42743 database.

S2. Validation of the predictive value of HOXB-AS4 in HNSCC using external datasets. (A-B) Kaplan-Meier survival analysis of HOXB-AS4 in HNSCC between high- and low-risk groups in GSE41613 and GSE42743 database.

S3. GO and KEGG enrichment analysis were carried out in HOXB-AS4 high-low expression subgroups, and the results were confirmed by cellular immunofluorescence. (A) GO enrichment analysis. (B) KEGG enrichment analysis. (C) Cytoskeletal changes were observed by cellular immunofluorescence

Supplementary Figures

Supplementary Fig.1

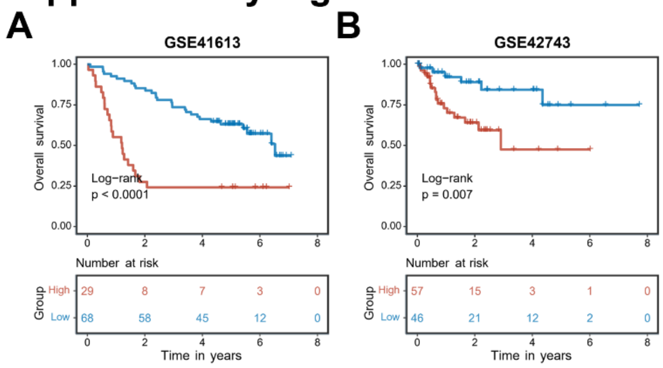

Supplementary Fig.2

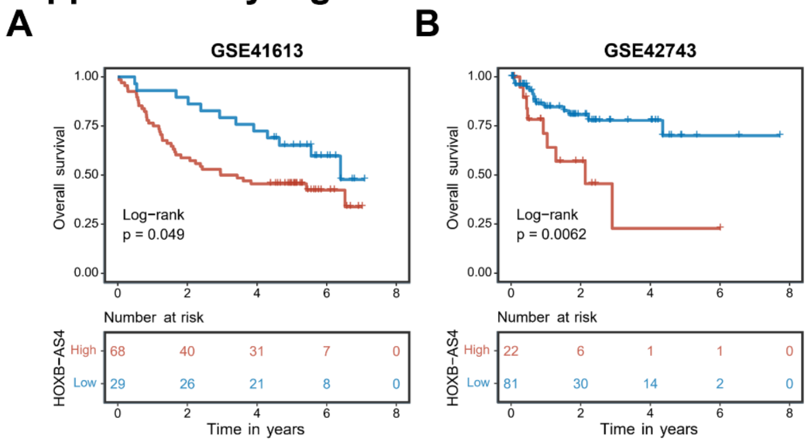

Supplementary Fig.3

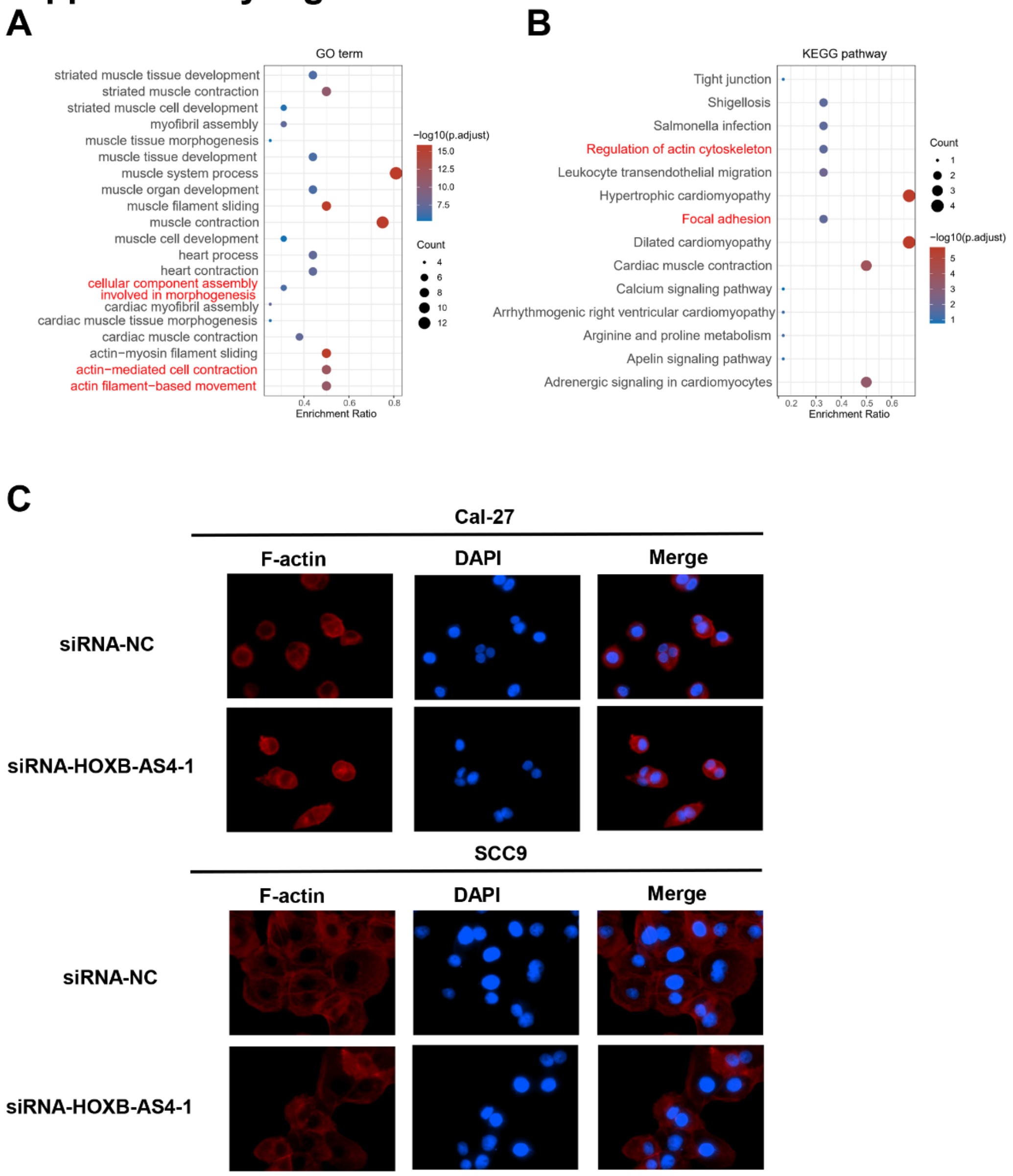

Supplement: Supplementary file 1 — Supplementary figures. [file jcav16p2997s1.pdf]
